# Use of Light Sensor and Focused Local Atrial Electrogram Recordings for the Monitoring of Thermal Injury to the Esophagus and Lungs During Laser Catheter Ablation of the Posterior Atrial Walls: Preclinical In Vitro Porcine and In Vivo Canine Experimental Studies

**DOI:** 10.19102/icrm.2019.100703

**Published:** 2019-07-15

**Authors:** Helmut P. Weber, Peter Schaur, Michaela Sagerer-Gerhardt

**Affiliations:** ^1^Section of Research and Development, CCEP Centre Taufkirchen, Taufkirchen, Germany; ^2^Hamamatsu Photonics, Herrsching, Germany

**Keywords:** Atrial fibrillation, esophageal thermal injury, focused local atrial electrograms, laser catheter ablation, laser light sensor

## Abstract

During the catheter ablation of atrial fibrillation, thermal damages to the esophagus may have deleterious effects. The use of the SensoLas light sensor (SLLS; LasCor GmbH, Taufkirchen, Germany) and focused local atrial electrograms (LEGs) were tested as means for the assessment of thermal effects on the esophagus during laser catheter ablation. A total of 32 transcatheter in vitro and in vivo 1064-nm laser impacts were aimed at porcine (n = 16) and canine (n = 16) atrial endocardia. Photons scattering through the atrial and esophageal walls were captured by the SLLS, transmitted via an optical fiber to a diode, and converted to power displayed on a monitor. The laser was stopped automatically when the power measurement reached values beyond the preset upper limit. During in vivo laser applications, bipolar LEGs were recorded via the miniature electrodes of the laser catheter. Thermal damage to the esophagus was avoided when the power measurement was limited to 150 μW or less and the diode current was 60 μA or less, regardless of the energy setting used and regardless of the thicknesses of the atrial and esophageal walls. Laser energy applied for eight seconds to 13 seconds (average: 10 seconds) abolished the electrical potentials permanently. In conclusion, the control of laser light via the SLLS and of atrial potential amplitudes in the LEGs can prevent thermal esophageal and lung injury during laser catheter ablation.

## Introduction

Catheter ablation for atrial fibrillation (AF) is a useful treatment option and has been offered as first-line therapy.^1^ However, collateral damage to adjacent structures has previously been described,^2–6^ including the formation of left atrioesophageal fistula, a rare but often fatal complication of catheter ablation for AF.^7^

Several methods have been proposed to date for detecting and avoiding esophageal injury during left atrial (LA) catheter ablation, including fluoroscopic contrast visualization of the esophagus during the procedure^8^ and temperature monitoring using various temperature probes.^9,10^ Separately, active techniques for esophageal protection have been proposed, including mechanical deflection of the esophagus,^11^ active esophageal cooling,^12^ and inflating an intrapericardial balloon between the LA and the esophagus^13^ in order to prevent esophageal injury during ablation of the posterior LA wall. However, owing to their limited success and laborious application, none of these approaches have been widely used. Esophageal temperature control remains explored only in a limited fashion and most experts advise to simply curb energy delivery near the esophagus because “what you cannot measure, you cannot control.”^14^ Here, we describe novel techniques that may allow for the control of thermal effects on the esophagus and lungs and could help to prevent collateral effects on mediastinal structures including esophageal thermal injury during laser ablation.

## Methods

**Figure 1:**
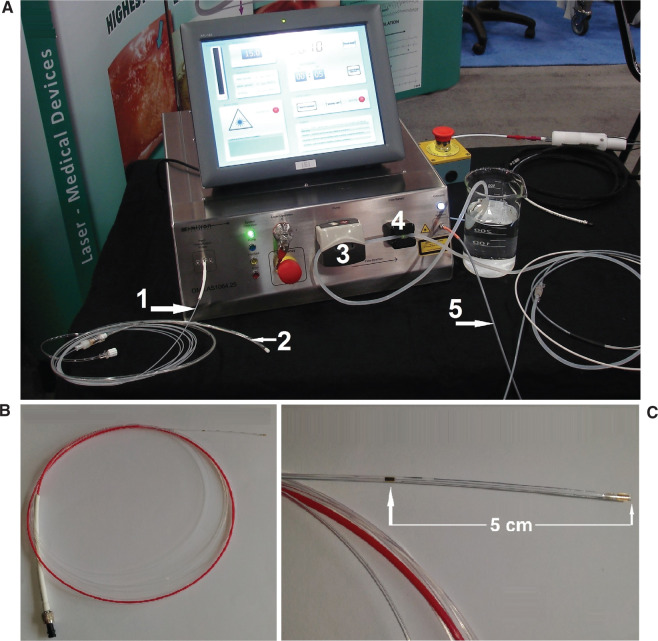
**A:** Frontal view of the custom-made, 1064-nm MediLas D diode laser (LasCor GmbH, Taufkirchen, Germany) with (1) the optical fiber of the SLLS and (2) its distal end with the light sensor fed into a transparent esophageal probe. Additionally, (3) the roller-pump for saline irrigation of the RLC, (4) ultrasound flow control of catheter irrigation, and (5) the open-irrigated ELMA RLC itself are indicated. **B:** Overview of the transesophageal SLLS catheter. **C:** The SLLS catheter’s distal end containing the 5-cm-long light sensor with X-ray markers.

The SensoLas light sensor (SLLS; LasCor GmbH, Taufkirchen, Germany)^15^ is a 3-French flexible optical fiber with a core diameter of 400 μm and an overall fiber length of 3 m that was connected at its proximal end via a subminiature version A (SMA 905) connector to the power source, which was a custom-made, 1064-nm neodymium-doped yttrium aluminum garnet laser (MediLas; LasCor GmbH, Taufkirchen, Germany) **(Figure 1)**. The active sensor length used was 5 cm. Its function is based on the principle of the integrating sphere, the Ulbricht sphere.^16^ The sensor collects the laser light, which is spread diffusely into the mediastinum during the application of laser energy aimed at the posterior LA wall. The sensor is visible on both computed tomography and X-ray scans due to radiomarkers and is compatible with magnetic resonance imaging technology. For measurement of the light, the distal segment with the sensor is introduced in a transparent uncolored transesophageal probe positioned with its sensing segment behind the LA cavity. Cumulative photon counting was performed by means of a special diode (G8371; Hamamatsu Photonics, Hamamatsu City, Japan) with a sensitivity of 0.4 A/W (0.4 μA/μW). The laser was stopped automatically by a signal of the photon counter in the case of exceeding the preset upper limits of power. The open-irrigated, bipolar electrode–laser mapping and ablation (ELMA) RytmoLas catheter (RLC; LasCor GmbH, Taufkirchen, Germany) is described in detail elsewhere.^17^ Saline irrigation of the RLC was performed by means of a peristaltic pump integrated in the laser and designed for the controlled delivery of liquid irrigation media. Saline flow was controlled via a foot switch that augmented the continuous flow automatically from 15 mL/min to 30 mL/min with the start of the laser.

**Figure 2:**
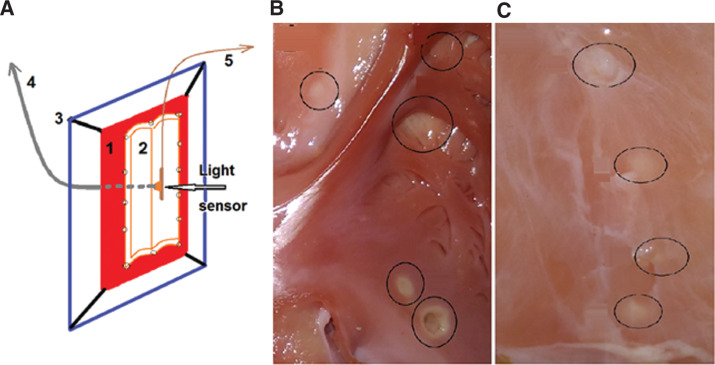
**A:** (1) The opened porcine posterior atrial walls; (2) the opened distal porcine esophagus, sutured on the epicardial side of the atria; (3) both atrial walls expanded vertically on a rectangular frame; and (4) the RLC with its tip in intimate contact with and oriented perpendicularly toward the endocardial atrial surface and with its opposite proximal end positioned to the laser (upper left arrow). During laser application, the light was oriented to the sensor (horizontal arrow) positioned vertically and opposite to the light spot emanated from the end hole of the RLC and was connected proximally to the current- and power-measuring diode (5) (upper right arrow). **B:** Endocardial view showing the laser lesions as pale circular spots (black circles), produced by laser application of various energy settings aimed at selected areas of the posterior atrial walls. **C:** View of the outer side of the esophageal wall showing four pale spots of coagulation necrosis (black circles) measuring 3 mm to 4 mm in diameter and produced after transmural coagulation of the atrial wall.

In a first step of this project, in vitro experiments were carried out. These were performed at the ZPF Center for Preclinical Research of the Technical University of Munich in Munich, Germany, on a porcine heart specimen harvested from an animal following another experiment in the same institution. The atria were separated from the ventricles, opened, and stretched on a frame. On the epicardial side, a longitudinally opened segment of the esophagus from the same animal was sutured with its outer surface in contact with the epicardium. With the so-prepared experimental setting and the frame positioned vertically, laser applications were aimed at the endocardial surface, whereas the laser sensor was fixed directly opposite to the area illuminated by the helium-neon pilot laser. After a total of 16 laser applications of various energy settings aimed at selected endocardial sites, the esophagus was separated from the epicardial surface and inspected for thermal lesions. Spots of pale coagulation necrosis produced in the esophagus by way of transmural heating through the atrial wall were measured and documented **(Figure 2)**.

In a second step, in vivo animal experiments in mongrel dogs were performed at the Heart Rhythm Institute and Department of Medicine, University of Oklahoma in Oklahoma City, OK, USA. The animal experiments complied with the principles outlined in the Declaration of Helsinki. Animal experimental studies conformed to the *Directive 86/609/EEC on the Protection of Animals Used for Experimental and Other Scientific Purposes*, adopted in 1986 by the European Commission, and with *Laws and Regulations and Guidelines of the United States Food and Drug Administration Regarding Good Laboratory Practice and Nonclinical Research (CFR Title 21, Parts 11 and 58).*

Dogs were anesthetized by intravenous thiamylal-Na 4%, 0.4 mL/kg and intubated with isoflurane 0.8% to 1.5% and nitrous-oxide anesthesia. Catheterization was performed pervenously from the right groin (Seldinger technique). The RLC was inserted via a steerable Agilis NxT™ sheath (Abbott Laboratories, Chicago, IL, USA) and was manipulated in the dog hearts under X-ray control during continuous bipolar focused local electrogram (LEG) recordings via the miniature electrodes on the tip of the RLC. Left heart access was achieved using side-selective transseptal laser puncture procedures, as described elsewhere.^18^

**Figure 3:**
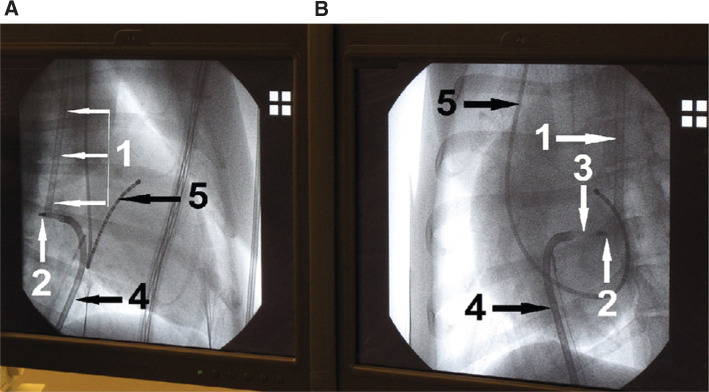
From left to right: right anterior oblique and left anterior oblique X-ray images of a dog heart during laser application aimed at the posterior left atrial wall showing (1) the SLLS catheter fed into a transparent esophageal probe and positioned with its sensing segment behind the posterior left atrial wall; (2) the tip of the RLC during its orientation to the esophagus; (3) the distal segment of the RLC beyond the end hole of the Agilis NxT™ sheath (Abbott Laboratories, Chicago, IL, USA); (4) the course of the aforementioned sheath; and (5) a multipolar electrode catheter from the subclavian vein positioned in the coronary sinus.

The SLLS was inserted via a transparent uncolored esophageal probe and its sensing distal segment was positioned under X-ray control behind the left atrium. For laser applications aimed at the posterior LA wall, the RLC was oriented with its tip perpendicularly towards the posterior atrial wall pointing directly to or closely to the vicinity of the sensor visible on the left anterior oblique axis or right anterior oblique axis of the X-ray images **(Figure 3)**.

The photon counter was adjusted for a stop of the laser when the maximum diode current measured was 60 μA or more and the maximum power measured was 150 μW or more, respectively. A total of 16 laser applications at various energy settings were aimed at selected sides of the posterior walls of the right and left atria (n = 8 each side) in two dogs. Between three hours and five hours after the experiments, the hearts and lungs were removed, and the lesions were evaluated morphometrically.

## Results

The minimal energy that produced thermal damage to the esophagus in vitro was 100 J; the diode currents thereby measured were 64 μA and 92 μA and power values were 160 μW and 230 μW, respectively. Esophageal lesions were conspicuous also after applications of 200 J and 400 J. However, maximum diode current and maximum power values measured here were somewhat less than those during the application of 100 J, totaling 57 μA and 74 μA and 142 μW or 185 μW, respectively **(Table 1)**. None of the other applications produced esophageal lesions, regardless of the amount of energy applied.

**Figure 4:**
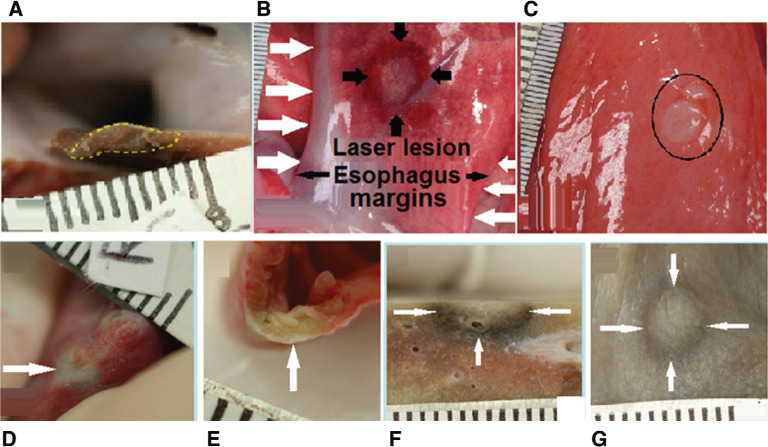
**A:** Cross-section through the left atrial posterior wall of a dog heart showing a three-hours-old transmural lesion of coagulation necrosis (yellow dotted line) produced by laser application (15 W for 30 seconds with catheter saline irrigation flow of 35 mL/min), with a depth of 2.0 mm and a diameter of 6.8 mm and with accompanying collateral thermal damage to the esophagus. **B:** Superficial view of the esophagus showing its margins (horizontal white arrows) and a pale spot surrounded by a circular hemorrhage. The lesion diameter was 16.0 mm (margins are shown by thick black arrows). **C:** Inner view of the lesion with a pale spot of transmural coagulation necrosis at a diameter of 8.0 mm and a small separate oval spot of 2.0 mm × 2.5 mm at its top (at one o’clock, within the black oval). **D:** Epicardial view of a right atrial lesion produced by laser application (15 W for 10 seconds) showing a pale spot with a diameter of 3.2 mm (horizontal white arrow). **E:** Transmural lesion depth was 1.5 mm (vertical white arrow). **F**: Collateral lung lesion at a depth of 4.0 mm. **G:** Surface view of the oval 6.5 mm × 4.5 mm lung lesion (margins are indicated by white arrows).

Based on the in vitro experiences, for the in vivo experiments, the laser was programmed to stop when the diode current reached 60 μA and the power reached 150 μW, respectively. With this, four consecutive laser impacts were aimed at the LA posterior wall. In order to avoid overlapping of lesions, the catheter tip was repositioned after each application along the sensor axis. Posterior LA walls were found to be coagulated transmurally without thermal injury to the esophagus when the level of energy applied was limited to 150 J. Energy applications of 75 J or less did not produce an effect on the atrial walls **(Table 2)**. In contrast, however, when the laser stop was inactivated, a laser application of 300 J or more produced lesions of coagulation necrosis in the anterior esophageal wall, while a laser application of 150 J or more aimed at the right atrial wall produced lung injuries **(Figure 4).** Thus, a high diode current of more than 60 μA and a power level of more than 150 μW were measured.

**Figure 5:**
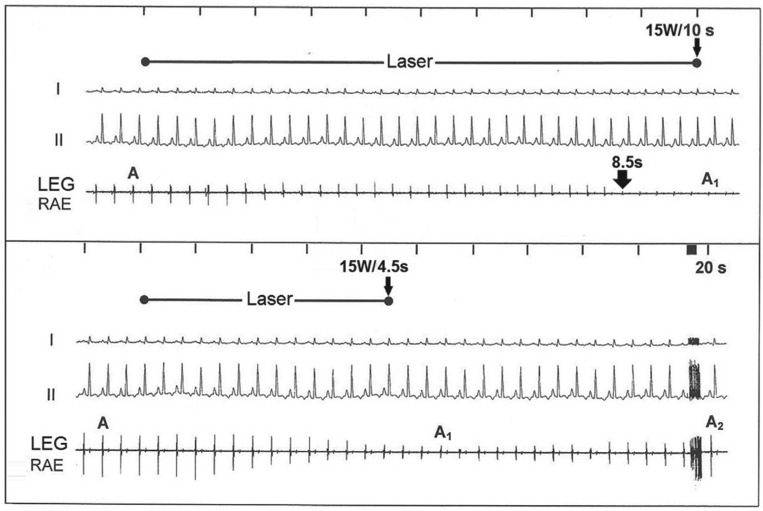
Bipolar focused right LEGs recoded via the tip electrodes of the RLC during laser application at 15 W were aimed at the posterior right atrial wall. On top, gradual abatement of local electrical potential amplitudes from A to A1 during 4.5 seconds of laser application, and subsequent recovery after 20 seconds (A1 to A2). On bottom, permanent abolishment of atrial electrical potential amplitudes after 8.5 seconds of laser application. I, II: surface lead electrograms.

**Figure 6:**
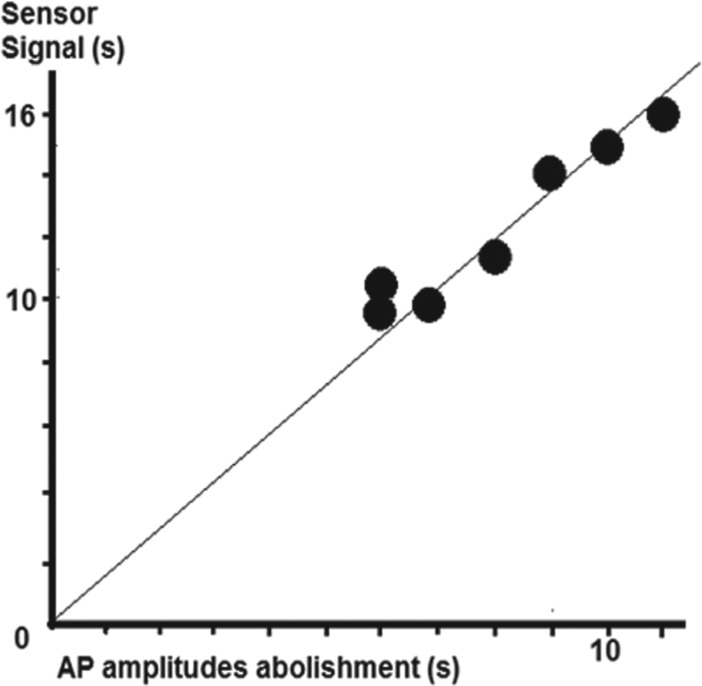
Correlation coefficient (near 1.0) between atrial potential abolishment and sensor signal when the distal sensing segment of the intraluminal esophageal SLLS was positioned behind the LA area where the catheter-directed laser light was aimed at.

Continuous LEG recordings via the miniature electrodes of the RLC during laser application showed a gradual abatement of local electrical potential amplitudes. Laser impacts of five seconds or less allowed for complete recovery of local electrical potential amplitudes and no atrial lesions were found in the targeted atrial region. During laser applications of more than five seconds, local electrical potential amplitudes dwindled further until their permanent abolishment after 10 seconds to 20 seconds **(Figure 5)**. In general, the permanent abolishment of local atrial potential amplitudes was achieved at three seconds to five seconds prior to the automatic stop of the laser or of the light-up signal of the inactivated laser stop of the photon counter **(Table 3 and Figure 6)**.

## Discussion

In this research, it was shown that, during laser catheter application aimed at the posterior LA wall, the thermal effects on the esophagus can be controlled by using the SLLS connected with a cumulative photon counter. By limiting the energy level below the coagulation threshold, thermal injury of the esophagus can be reliably avoided. The cumulative photon counter will always emit a stop signal to the laser when the preset upper photon limit is reached. This upper limit is chosen well below the temperature threshold of esophageal wall coagulation. Thus, a delayed dynamic of thermal injury evolution in the esophageal wall can be ruled out.

LA posterior wall thicknesses may vary widely in patients. The midposterior and inferoposterior walls can measure 1.58 mm ± 0.22 mm and 1.74 mm ± 0.18 mm, respectively.^19^ Regardless, when the amount of photons captured within the esophageal lumen is approaching the preset upper limit, the laser energy application will be stopped automatically. Thus, laser application is stopped when overheating of the esophageal wall is imminent, prior to the occurrence of thermal lesion development. However, the protective effect comes down exclusively to the protection of the esophagus. Importantly, atrial wall transmural coagulation is always achieved prior to the stop of the laser by the SLLS. Thus, outcomes of the laser catheter ablation of arrhythmias including atrial fibrillation will not be jeopardized by the sensor-induced laser cessation.

Of critical importance is the observation that the transmurality of atrial lesions was always achieved prior to the laser stop by the sensor as reflected by the permanent abolition of potential amplitudes in the LEG. Strict correlation between the abatement of electrical potential amplitudes recorded via the miniature electrodes on the tip of the RLC during laser application may be applicable for the timing of laser applications in the right atrial areas in situations where photon sensing up to now has not been practicable.

The spread of heat in tissue is a gradual process and the thermal inertia of the tissue slows down the distribution process of heat. This is indicative of the observation that, with laser applications of less than five seconds, electrical potential amplitudes regain their initial heights and no lesion is produced in the culprit atrial area. For the distantly located esophagus, thermal damage can be ruled out. With the automatic laser stop occurring at three seconds to five seconds after the transmurality of atrial lesion photon scattering is stopped, heat distribution in the adjacent tissue does not occur or is minimal in nature and thermal effects if any are present are limited to reversible vasodilation, slight edema, and possibly modest hemorrhage. It can be anticipated that, by stopping laser application with the abolishment of local potential amplitudes, mediastinal structures such as the esophagus, lungs, and nerves, with special importance for the vagus,^20^ can be protected from thermal injury without jeopardizing ablation success.

Acute transmural atrial laser lesions of coagulation necrosis, once achieved, will result in a chronic scar that will be permanently devoid of electrical activity.^17,21–23^ The SLLS together with online control of electrical potential amplitudes in the focused LEG during laser application may help to protect all of the mediastinal structures from thermal injury and may substantially contribute to the safety and efficacy of the ablation procedure. The SLLS prevents further increase of the mediastinal temperature when overheating is imminent, prior to the occurrence of thermal damage to the esophagus. This is a special claim of the SLLS function when the laser method of ablation is applied. In contrast, it is unclear whether luminal esophageal temperature monitoring using insulated thermistor probes for radiofrequency LA ablation may help to reduce esophageal thermal damage.^24^ Moreover, the use of esophageal temperature probes in the setting of atrial fibrillation catheter ablation per se appears to be a risk factor for the development of endoscopically detected esophageal lesions.^25^

### Limitations

Importantly, the long-term nature of the lesions produced in this study is unknown, as the animals were sacrificed at three hours to five hours after lesion creation. In order to confirm our results, further studies with longer periods of postoperative observation are warranted.

## Conclusion

In our experience, the use of the SLLS is conceivably the best method available for the prevention of thermal injury to the esophagus so far. The described technique is practicable only when the laser ablation method is used. Control of electrical potential amplitudes with abolishment of potentials in the focused LEG recordings may help to avoid thermal injury to the mediastinal structures, provided that electrograms are recorded without noise during energy application.

## Figures and Tables

**Table 1:** Maximal Current and Power Measurements Obtained Using a G8371 Diode* during 1064-nm In Vitro Laser Application**

Laser Power Applied (W)	Radiation Time (s)	Energy Applied (J)	Maximum Diode Current Measured (μA)	p-value (μA)	Maximum Power Measured (μW)	p-value (μW)
5 W	5	25	18	23 ± 7.2 vs. 29 ± 21; p = 0.80 (NS)	45	59 ± 18 vs. 73 ± 53; p = 0.80 (NS)
	10	50	42		105	
	15	75	8		20	
	20	100	26		100	
			Mean: 23 ± 7.2		Mean: 59 ± 18	
10 W	5	50	0	29 ± 21 vs. 8 ± 2.8; p = 0.96 (NS)	0	73 ± 53 vs. 70 ± 7.0; p = 0.96 (NS)
	10	100	92 (+)		230 (+)	
	15	150	13		33	
	20	200	12		30	
			Mean: 29 ± 21		Mean: 73 ± 53	
15 W	5	75	28	28 ± 2.8 vs. 54 ± 11; p = 0.06 (NQS)	70	70 ± 7.0 vs. 136 ± 28; p = 0.06 (NQS)
	10	150	23		58	
	15	225	26		65	
	20	300	36		90	
			Mean: 28 ± 2.8		Mean: 70 ± 7.0	
20 W	5	100	64 (+)		160 (+)	
	10	200	57 (+)		142 (+)	
	15	300	23		57	
	20	400	74 (+)		185 (+)	
			Mean: 54 ± 11		Mean: 136 ± 28	
5 W	15	75	Min = 8 μA		Min = 20 μW	
10 W	10	100	Max = 92 μA		Max = 230 μW	

NS: not significant; NQS: not quite significant.

*Hamamatsu Photonics, Hamamatsu City, Japan.

**Laser applications occurred at various powers and application times (n = 4 each) aimed at selected sides of the exposed porcine right and left atrial endocardial surfaces and with the 5.0-cm-long SLLS placed in intimate contact along the opened inner esophageal wall during laser radiation using the open-irrigated RLC. The lesions achieved were circular white spots of coagulation necrosis measuring 1.0 mm to 2.0 mm deep with diameters of 3.0 mm to 4.0 mm conspicuous on the external esophageal surface. Diode current and power values thereby measured were 57 μA or more and 142 μW or more, respectively (+).

**Table 2:** Sizes of Transmural Atrial Lesions Following In Vivo Laser Application*

Application Times (s)/Energy Applied (J)	Atrial Lesions		Collateral Lesions	Depth (mm)	Diameter (mm)
	Depth (mm)	Diameter (mm)			
Left atria					
5/75	Ø	Ø	Present in the esophagus	Ø	Ø
10/150	1.0	3.0		Ø	Ø
10/150	1.5	3.3		Ø	Ø
10/150	1.0	3.3		Ø	Ø
20/300	4.2	5.2		1.0	3.0
20/300	4.2	5.7		Ø	Ø
30/450	2.0	6.8		Transmural	4.2
30/450	2.0	6.9		Transmural	5.0
	Mean: 1.9, SD: 1.5	Mean: 4.8, SD: 2.3			Mean: 4.1, SD: 1.0
Right atria					
5/75	Ø	Ø	Present in the lungs	Ø	Ø
10/150	1.5	3.2		4.0	6.5 × 4.5
10/150	2.0	5.0		4.2	10.0 × 7.0
15/225	2.5	5.0		3.0	7.0 × 4.5
15/225	4.3	5.2		Ø	Ø
20/300	4.5	5.7		Ø	Ø
20/300	7.2	8.3		Ø	Ø
30/450	4.2	2.6		3.7	9.0 × 7.5
	Mean: 2.8, SD: 0.75	Mean: 4.4, SD: 0.87		Mean: 3.7, SD: 0.3	Mean: 7.0, SD: 1.9

SD: standard deviation; Ø: no lesion.

*Laser applications were performed under X-ray control at a power of 15 W and various application times, aimed at the right atrial and LA posterior walls in dog hearts using the open-irrigated RLC.

**Table 3:** Time Differences Between the Permanent Abolishment of Atrial Potential Amplitudes* and the Alarm Signal of the Temperature Safety Chain

LA Lasing Time (s)	Permanent Abolishment of AP Amplitudes (s)	Sensor Signal (s)	Time Difference (s)
5	AP recurrences	Ø	–
10	6	10	+4
10	7	10	+3
10	6	10	+4
20	10	15	+5
20	11	16	+5
30	9	14	+5
30	8	11	+3
	Mean: 8.14 SD: 1.95	Mean: 12.3 SD: 2.63	Mean: 4.14
	p = 0.0058**		

AP: atrial potential; LA: left atrial; SD: standard deviation; Ø: no signal.

*As recorded in the focused local electrograms via the minielectrodes from the tip of the RLC during laser application.

**According to conventional criteria, this difference is considered to be very statistically significant.

## References

[1] Verma A, Natale A (2005). Should atrial fibrillation ablation be considered first-line therapy for some patients? Why atrial fibrillation ablation should be considered first-line therapy for some patients.. Circulation..

[2] Moennig G, Wessling J, Juergens KU (2005). Further evidence of a close anatomical relation between the esophagus and pulmonary veins.. Europace..

[3] Shah D, Dumonceau JM, Burri H (2005). Acute pyloric spasm and gastric hypomotility: an extracardiac adverse effect of percutaneous radiofrequency ablation for atrial fibrillation.. J Am Coll Cardiol..

[4] Aupperle H, Doll N, Walther T (2005). Ablation of atrial fibrillation and esophageal injury: effects of energy source and ablation technique.. J Thorac Cardiovasc Surg..

[5] Weber H, Sagerer-Gerhardt M (2014). Open-irrigated laser catheter ablation: relationship between level of energy, myocardial thickness, and collateral damages in a dog model.. Europace..

[6] Berjano EJ, Hornero F (2005). What affects esophageal injury during radiofrequency ablation of the left atrium? An engineering study based on finite-element analysis.. Physiol Meas.

[7] Schmidt M, Nolker G, Marschang H (2008). Incidence of esophageal wall injury post-pulmonary vein antrum isolation for treatment of patients with atrial fibrillation.. Europace..

[8] Yamane T, Matsuo S, Date T (2006). Visualization of the esophagus throughout left atrial catheter ablation for atrial fibrillation.. J Cardiovasc Electrophysiol..

[9] Cummings JE, Schweikert RA, Saliba WI (2005). Assessment of temperature, proximity, and course of the esophagus during radiofrequency ablation within the left atrium.. Circulation..

[10] Gianni C, Atoui M, Mohanty S, Trivedi Ch,  Bai R, Al-Ahmad A (2016). Difference in thermodynamics between two types of esophageal temperature probes: insights from an experimental study.. Heart Rhythm..

[11] Herweg B, Johnson N, Postler G (2006). Mechanical esophageal deflection during ablation of atrial fibrillation.. Pacing Clin Electrophysiol..

[12] Tsuchiya T, Ashikaga K, Nakagawa S (2007). Atrial fibrillation ablation with esophageal cooling with a cooled water-irrigated intraesophageal balloon: a pilot study.. J Cardiovasc Electrophysiol..

[13] Buch E, Nakagawa S, Shivkumar K (2008). Intra-pericardial balloon retraction of left atrium: a novel method to prevent esophageal injury during catheter ablation.. Heart Rhythm..

[14] Syed FF, Oral H (2016). Esophageal temperature and atrio-esophageal fistula: “If you cannot measure it, you cannot control it”.. Heart Rhythm..

[15] Weber H (2013). Laser safety device.

[16] Palmer JM, Barbara G, Grant BG (Press). The Art of Radiometry.

[17] Weber H, Schmitz L, Heinze A, Ruprecht L, Sagerer-Gerhardt M,  Bellucci C (2017). The development of a laser catheter with improved mapping resolution and online monitoring of lesion formation during arrhythmia ablation. Laser Ablation.

[18] Weber H, Sagerer-Gerhardt M (2013). Side-selective atrial transseptal laser puncture.. J Innov Cardiac Rhythm Manage..

[19] Hayashi H, Hayashi M, Miyauchi Y (2014). Left atrial wall thickness and outcomes of catheter ablation for atrial fibrillation in patients with hypertrophic cardiomyopathy.. J Interv Card Electrophysiol..

[20] Jacobs V, May HT, Crendall BG, Ballantyne B, Chisum B, Johnson D (2018). Vagus nerve injury symptoms after catheter ablation for atrial fibrillation.. Pacing Clin Electrophysiol..

[21] Weber H, Sagerer-Gerhardt M (2015). Monitoring of laser effects on the conduction system by using an open-irrigated electrode-laser mapping and ablation catheter; laser catheter mapping.. Europace..

[22] Weber H, Sagerer-Gerhardt M (2014). Electrocardiographic monitoring of myocardial lesion formation during laser catheter ablation in a dog model.. J Innov Cardiac Rhythm Manage..

[23] Weber H, Heinze A, Ruprecht L, Sagerer-Gerhardt M (2018). Laser catheter modulation of the sinus node in the treatment of inappropriate sinus tachycardia: experimental and clinical results.. J Innov Cardiac Rhythm Manage..

[24] Halbfass P, Müller P, Nentwich K (2017). Incidence of asymptomatic esophageal lesions after atrial fibrillation ablation using an esophageal temperature probe with insulated thermocouples: a comparative controlled study.. Europace..

[25] Müller P, Dietrich JW, Halbfass P (2015). Higher incidence of esophageal lesions after ablation of atrial fibrillation related to the use of esophageal temperature probes.. Heart Rhythm..

